# A Proteomic Characterization of *Bordetella pertussis* Clinical Isolates Associated with a California State Pertussis Outbreak

**DOI:** 10.1155/2015/536537

**Published:** 2015-05-24

**Authors:** Yulanda M. Williamson, Hercules Moura, Jennifer Whitmon, Adrian R. Woolfitt, David M. Schieltz, Jon C. Rees, Stephanie Guo, Heather Kirkham, Daniel Bouck, Edwin W. Ades, Maria Lucia Tondella, George M. Carlone, Jacquelyn S. Sampson, John R. Barr

**Affiliations:** ^1^Division of Laboratory Sciences, National Center for Environmental Health, Centers for Disease Control and Prevention, Chamblee, GA 30341, USA; ^2^Division of Bacterial Diseases, National Center for Immunizations and Respiratory Diseases, Centers for Disease Control and Prevention, Atlanta, GA 30333, USA; ^3^Oak Ridge Institute for Science and Education, Oak Ridge, TN 37831-0117, USA

## Abstract

*Bordetella pertussis* (*Bp*) is the etiologic agent of pertussis (whooping cough), a highly communicable infection. Although pertussis is vaccine preventable, in recent years there has been increased incidence, despite high vaccine coverage. Possible reasons for the rise in cases include the following: *Bp* strain adaptation, waning vaccine immunity, increased surveillance, and improved clinical diagnostics. A pertussis outbreak impacted California (USA) in 2010; children and preadolescents were the most affected but the burden of disease fell mainly on infants. To identify protein biomarkers associated with this pertussis outbreak, we report a whole cellular protein characterization of six *Bp* isolates plus the pertussis acellular vaccine strain *Bp* Tohama I (T), utilizing gel-free proteomics-based mass spectrometry (MS). MS/MS tryptic peptide detection and protein database searching combined with western blot analysis revealed three *Bp* isolates in this study had markedly reduced detection of pertactin (Prn), a subunit of pertussis acellular vaccines. Additionally, antibody affinity capture technologies were implemented using anti-*Bp* T rabbit polyclonal antisera and whole cellular proteins to identify putative immunogens. Proteome profiling could shed light on pathogenesis and potentially lay the foundation for reduced infection transmission strategies and improved clinical diagnostics.

## 1. Introduction

*Bordetella pertussis* (*Bp*) is the causative agent of pertussis (whooping cough), a highly transmissible bacterial respiratory infection [1, 2]. Although pertussis is a vaccine-preventable disease and vaccine coverage is high within developed countries, increased incidence has been reported among all populations, with marked increases in adolescents and adults. Even more, infants aged <1 year, who are at greatest risk for severe disease and death, continue to account for a significant share of the reported cases [3]. In the United States (US), in 2010, there were nearly 28,000 reported cases of pertussis and, in 2012, 48,277 cases were reported to the CDC [4]. Included in these case numbers were several pertussis outbreaks which had occurred across different regions in the US. The greatest burden of disease in the 2010 California (CA) outbreak fell upon infants whom had not yet received the full immunoprotective benefits the acellular pertussis vaccine has to offer; however, there were still reported cases among traditional vaccinated-covered populations, such as children and adolescents [5, 6].

Several hypotheses have been suggested to explain the resurgence of pertussis, particularly in the US. They include pathogen adaptation, waning pertussis vaccine immunity, improved clinical diagnostics, and better surveillance [7–9]. In general, the rise in pertussis cases has led researchers to enhance pertussis surveillance efforts, biologically examine recent circulating* Bp* strains, and reevaluate current pertussis vaccination strategies as a means to gain insight in the reported case increase. In the US, acellular pertussis vaccines (DTaP) used consist of five* Bp* proteins, inactivated pertussis toxin (Ptx), filamentous hemagglutinin (FHA), pertactin (Prn), and fimbriae serotype 2 and/or 3 (Fim2/Fim3) which have been purified from the historic* Bp* strains, Tohama I (T) or 10536 [10–12]. Recently, studies focusing on pathogen adaptation have reported genetic differences between the current circulating* Bp* strains and vaccine strains [8, 13–16] in genes encoding the five vaccine proteins. This genetic adaptation may have implications for bacterial protein expression, virulence, microbial pathogenesis, and more importantly vaccine-induced pertussis immunity. Thus, studies ascertaining differences in* B. pertussis* strain populations not only at the genomic level but also at the expressed protein level could help provide insight into the problem of pertussis resurgence.

Over the years, researchers have implemented traditional proteomic approaches in parallel with mass spectrometry (i.e., liquid chromatography-tandem mass spectrometry [LC-MS/MS]) to examine total proteins or protein subsets of clinical pathogens, such as* Bp* [17–21]. In this report, whole cellular protein profiles using gel-free LC-MS/MS were compiled on six 2010 CA* Bp* outbreak (CA2010) isolates compared to a well-characterized acellular pertussis vaccine strain,* Bp* T. Additionally, antibody affinity capture was performed using* Bp* whole cell protein extracts and rabbit polyclonal antisera generated against intact* Bp* T whole cell bacteria to assess the immunoreactivity profiles of the CA2010 isolates. Information gained from this proteomic study can aid in* Bp* strain differentiation associated with this outbreak, advance surveillance efforts for future pertussis outbreaks, and if necessary lay the groundwork for improved pertussis vaccine design.

## 2. Materials and Methods

### 2.1. Reagents

All reagents and media not vendor-specified were prepared at the CDC Core Facility. In addition, chemicals used in experimentation were obtained from either Sigma-Aldrich Chemical Company (St. Louis, MO, USA) or Fisher Scientific (Pittsburgh, PA, USA) as noted.

### 2.2. Bacterial Strains

**Figure 1:**
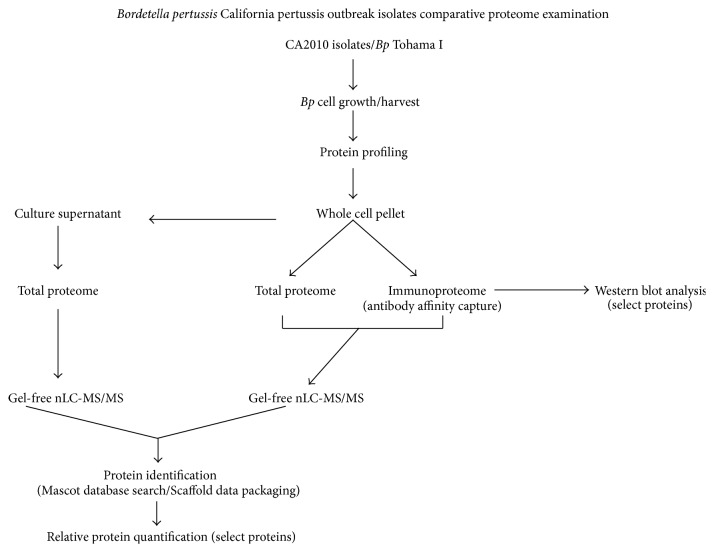
A flow diagram schematically summating the core methodologies of the* B. pertussis* California pertussis outbreak isolates comparative proteomic examination.

Seven* Bp* strains, Tohama I (T), and six CA2010 clinical outbreak isolates were used in this study. T, first isolated in Japan in 1954, is a well-characterized and completely genome sequenced* Bp* strain that has been used as the basis of vaccines in many countries for several years [22]. It is characterized as type II by pulse-field gel electrophoresis (PFGE) analysis and possesses the pertactin I (*prn1*) and pertussis toxin (*ptxA2*) genotype typical of prevaccine era isolates [22–24]. The CA2010 isolates used in the study were initially selected as part of a collaborative project between the CA state Department of Public Health and other scientists in the pertussis research community. (This working group's aim was to determine the phylogenetic relationship of* Bp* isolates from CA children stratified by severity of illness. These CA2010 strains were originally isolated from infants less than 3 months of age, in which* Bp* infection led to either fatal or nonfatal pertussis disease.) The strains were received by the CDC Pertussis & Diphtheria Laboratory and further shared with the CDC Biological Mass Spectrometry Laboratory (BMSL) for proteomic characterization. It is worth mentioning that since the CA2010 isolates shared with the group had not been genetically sequenced, the intent was to use the historic acellular vaccine strain (*Bp* T) to comparatively profile protein expression versus the contemporary CA2010 isolates. Even more, the* Bp* strain designation assigned by the CA state health department CA2010-1 through CA2010-12 was changed for simplicity by the BMSL and are noted as* Bp* 1 through* Bp* 12, respectively. The various proteomic approaches utilized are summarized in the flow chart (Figure 1).

### 2.3. Bacterial Cell Culture

All* Bp* strains were plated on Bordet-Gengou agar and incubated at 35°C with 5% CO_2_ for 4 days [25]. The bacteria were subsequently subcultured into Modified Stainer-Scholte (MSS) media at 35°C, with aeration at 200 rpm in a Beckman-Coulter shaker (Beckman-Coulter, Brea, CA) until comparable midlog growth phases (optical densities, OD_600_ between 0.7 and 1.0) were reached. The bacterial strains were then pelleted from MSS by centrifugation at 8,000 ×g for 30 min at 4°C and the culture supernatant (CS) retained. The pellets (1 × 10^9^ cells based on visual turbidimetric measurements) were washed two times in distilled water (dH_2_O) and stored at −70°C for further use. All strains were cultured and grown three separate times.

### 2.4. *Bp* Protein Extraction

Whole cellular soluble protein fraction extracts (WCF) used for total proteome and immunoproteome analysis were prepared as described by Bruker Daltonics [26]. Briefly for each isolate, whole cell bacteria pellets were resuspended in sterile DI water followed by the addition of 100% ethanol and centrifuged at 12,000 ×g for 2 minutes at room temperature (RT). The supernatant was removed and the pellet was dissolved in 70% formic acid followed by the addition of 100% acetonitrile. The suspension was centrifuged at 12,000 ×g for 2 minutes at RT and the protein extract was stored at −70°C until further use. For proteome analysis of the culture supernatants (CS), 10 mL of each supernatant was vacuum centrifuged using a refrigerated centrivap concentrator (Labconco, Kansas City, MO) to concentrate the sample by roughly a factor of two, followed by trichloroacetic acid with acetone protein precipitation [27]. All protein concentrations were determined using BSA standard quantitation (BCA, Pierce, Rockford, IL). Protein integrity was assessed via sodium dodecyl sulfate-polyacrylamide gel electrophoresis (SDS-PAGE) (Invitrogen, Carlsbad, CA). To visualize separated proteins, gels were stained using gel-coded Coomassie or silver staining protocols.

### 2.5. *Bp* Whole Cell Protein Identification

Before direct proteolytic cleavage, protein fractions (WCF or CS) of* Bp* T,* Bp* 2, 4, 5, 9, 11, and 12 (10 *μ*g each) were dried completely via vacuum centrifugation and resolubilized with 0.1% Rapigest SF surfactant (RG, Waters Corporation, Milford, MA) in 50 mM NH_4_(CO_3_)_2_, 1 mM CaCl_2_, and pH 8.5 digestion buffer at 100°C for 5 min to denature proteins. Upon cooling at RT, the samples were incubated overnight (ON) with trypsin (10 *μ*g) (Promega Corporation, Madison, WI) at 37°C. After incubation, the RG was cleaved with 1 M HCl (final 175 mM) for 30 min at 37°C and centrifuged at 12,000 ×g for 15 min. The supernatant was removed and suspended in equal volumes of 0.1% formic acid and analyzed by nanoflow LC-electrospray ionization MS/MS (nLC-ESI-MS/MS). The protein profiles for each strain represent three distinct biological preparations, each analyzed in duplicate.

### 2.6. *Bp* Immune Sera

Three-week-old female rabbits were injected intraperitoneally (i.p.) with 1 × 10^9^ colony forming units (cfu) of* Bp* T suspended in 10 *μ*L of physiological saline (pH 7.2). Before injection, the strain was cobalt-irradiated using 5 × 10^6^ *γ* RAD to inhibit bacterial replication and infectivity, while preserving bacterial surface structures. The process was repeated every 2 weeks thereafter, with three separate i.p. immunizations of similar dosage for 6 weeks. At this time, rabbits were euthanized according to AALAC and IACUC standards and the* Bp* T immune sera generated was collected from blood. The collected serum was aliquoted and stored at −70°C until use. All experiments were performed in accordance with project specific animal protocol # 1642, “Production of Antibodies to* Bordetella pertussis* in New Zealand White Rabbits” approved by the Institutional Animal Care and Use Committee (IACUC), CDC.

### 2.7. Immunoprecipitation Studies Using Antibody Affinity Magnetic Bead Capture Technology

Dynabeads (Invitrogen) coated with protein G for immunoglobulin (IgG) capture of* Bp* proteins and subsequent immunoprecipitation (IP) were used as described previously [17]. Briefly,* Bp* T-generated rabbit antisera-conjugated beads, as well as normal rabbit sera conjugated and beads only controls (comprising a triset), were prepared. Each triset was used to separately capture putative antigens in the* Bp* T or each CA2010 isolates' (*Bp* 2, 4, 5, 9, 11, and 12) WCF (10 *μ*g). Antibody-bead protein complexes were washed extensively prior to 0.1% RG treatment and overnight trypsin digestion (10 *μ*g). After digestion, each IP complex was magnetically stabilized, and the supernatant containing WCF tryptic peptides was transferred to a fresh tube and dried via vacuum centrifugation to concentrate the samples. The RG was inactivated and the samples prepared for nLC-ESI-MS/MS, in which peptides were suspended in equal volumes of 0.1% formic acid. The data represent three biological preparations, each analyzed in duplicate.

### 2.8. Western Blot Analysis

To confirm lack of protein detection, an unstained duplicate gel consisting of separated WCF from* Bp* T,* Bp* 2, 4, 5, 9, 11, and 12 (10 *μ*g each) as well as purified recombinant protein controls for western blots (i.e., Prn, Fim2, or Fim3) (Alpha Diagnostics International, San Antonio, TX) were electroblotted using the iBlot dry blotting system (Life Technologies). The protein-immobilized PVDF blot was subsequently immunodetected separately with either rabbit Anti-*Bp* Prn, Fim2, or Fim3 protein antiserum (Alpha Diagnostics International), using the anti-rabbit Westernbreeze chromogenic kit (Life Technologies).

### 2.9. Nanoflow Liquid Chromatography-Electrospray Ionization-Mass Spectrometry

Nanoflow liquid chromatography (nano-LC) was coupled with data-dependent tandem mass spectrometry and database searching as described in Williamson et al. [17]. A Velos-Orbitrap hybrid tandem mass spectrometer (Thermo Scientific, San Jose, CA) was programmed to perform data-dependent acquisition by scanning a mass-to-charge (*m*/*z*) range of 400 to 1600 at a resolution setting of 60,000 for precursor ion acquisition. For MS/MS analysis the mass spectrometer was programmed to select the top 15 most intense ions with two or more charges.

Mascot Distiller (Matrix Science, London, UK; version 2.2.1.0) was used to extract all tandem mass spectra from the Xcalibur. RAW files were subsequently searched using Mascot (version 2.2.0). The Mascot search parameters were set to use the entire NCBInr database or a modified subset of the NCBInr database to create a* Bordetella* specific subset based on the keywords “*Bordetella pertussis* Tohama I,” recognized proteins in which trypsin is used as the digestion agent. Additional parameters for the search were the inclusion of two missed cleavages, a fragment ion tolerance mass of 0.50 Da, and a precursor ion tolerance of 200 ppm, and oxidation was selected as a variable modification.

Scaffold (version Scaffold_4.0.6.1, Proteome Software, Portland, OR, US) was used to validate MS/MS based peptide and protein identifications. Peptide identifications were accepted if they could be established at greater than 95.0% probability as specified by the Peptide Prophet algorithm [28]. Protein identifications were accepted if they could be established at greater than 99.0% probability and contained at least two identified peptides [29]. With these stringent parameters of Peptide Prophet and Protein Prophet within the Scaffold software, the probability of a wrong assignment is below 0.1%. Genbank GI accession and gene numbers were provided by the National Center for Biotechnology Information (NCBI) http://www.ncbi.nlm.nih.gov/. PSORTb subcellular scores were used to predict and localize identified WCF or CS proteins (http://www.psort.org/psortb/) [30]. Also, KEGG identifiers using NCBI GI accession numbers were employed to assign functions to each of the identified proteins http://www.genome.jp/kegg/kegg3.html [19]. Lastly, Scaffold semiquantitative normalized values based on spectral counting were used as a measure of relative abundance [31, 32].

## 3. Results

### 3.1. CA2010 Whole Cellular Protein Profiling by nLC-MS/MS

Gel-free direct enzymatic (trypsin) qualitative protein identification was performed on* Bp* T and CA2010 isolates* Bp* 2, 4, 5, 9, 11, and 12. nLC-ESI-MS/MS of generated tryptic peptides followed by database search analysis identified a total of 1363* Bp* proteins among all 6 CA2010 isolates and* Bp* T. Furthermore, based on greater than 95% Scaffold (protein prophet) protein identification probabilities, total WCF proteins identified for each strain were as follows: 1117 (*Bp* T), 1166 (*Bp* 2), 1106 (*Bp* 4), 1056 (*Bp* 5), 1053 (*Bp* 9), 1059 (*Bp*11), and 1061 (*Bp* 12) (Table 1). Nearly 75% of all proteins identified were commonly expressed between* Bp* T and CA2010 isolates, while 22% were detected in various strain combinations, meaning present in at least two isolates and absent in the remaining. Even more, 2% of proteins identified were detected only in the CA2010 isolates versus* Bp* T, while nearly 1% of the proteomes constituted proteins unique to specific strains.

**Figure 2:**
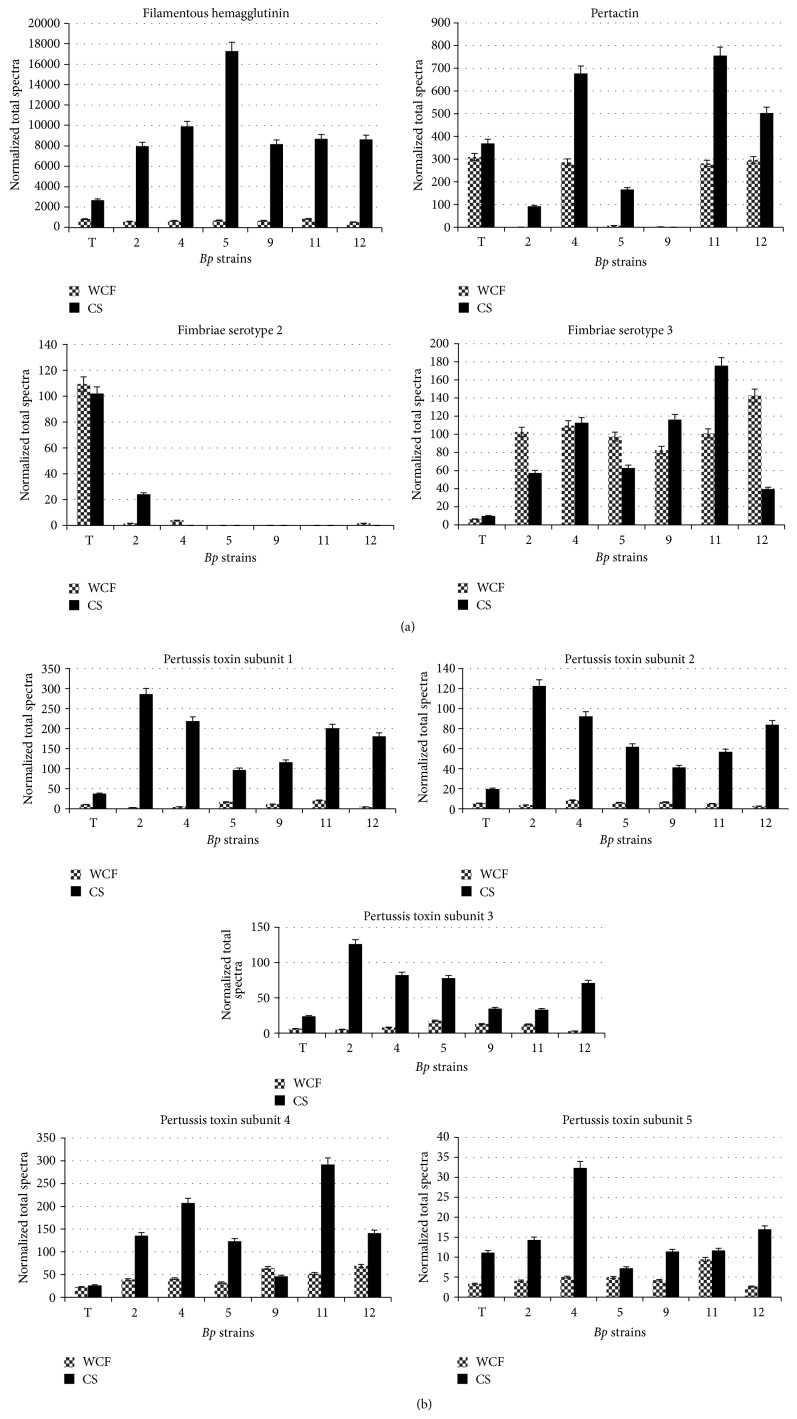
Comparative relative protein abundance profiles for* B. pertussis* acellular vaccine components. A bar chart illustration to compare normalized total spectra values for each acellular pertussis vaccine component identified in whole cellular fractions (WCF) (diamond bar) versus culture supernatant (CS) (black bar). (a) Filamentous hemagglutinin, pertactin, and serotype fimbriae 2/and 3. (b) Pertussis toxin subunits 1 through 5.

Based on PSORTb predictive scores over 50% of all the proteins profiled in the WCFs were localized to the cytoplasm, while 17% collectively made up cytoplasmic membrane, periplasmic, outer membrane, or extracellular-localized (surface-exposed or secreted) proteins with the remaining 30% of unknown location (see Figure S1a in Supplementary Material available online at http://dx.doi.org/10.1155/2015/536537). As determined by gene ontology tools, 70% of the proteins profiled were linked with cellular housekeeping functions such as cell growth, metabolism, DNA synthesis, transcription, translation, and transport, while the remaining 5% constitute proteins associated with adhesion, virulence, and pathogenesis, with nearly 25% identified having an unknown function (Figure S1b). Included among the 810 proteins commonly identified in all seven strains were three of the five possible acellular pertussis vaccine components (the 5-subunit Ptx (S1, S2, S3, S4, and S5), FHA, and Fim3). Other known* Bp* proteins, such as the Bvg virulence regulatory expression system proteins (Bvg A, Bvg S, and Bvg R), autotransporter Vag8, tracheal colonization factor (TcfA), periplasmic pertussis toxin assembly DsbA protein, outer membrane proteins A, P, and Q (OmpA, OmpP, and OmpQ), and adenylate cyclase/hemolysin (CyaA), were all identified in each WCF. However, the two remaining possible vaccine components, Prn and serotype Fim2, as well as the primarily extracellular dermonecrotic toxin (Dnt), were not detected in all of the strains. In addition, 27 proteins were identified only in the CA2010 isolates including a sulfate binding protein and members of the pertussis toxin liberating (Ptl) transport machineries, the latter associated with Ptx secretion. A combined total of 96 proteins were identified only in a specific strain, in which* Bp* T and* Bp* 2 had the greatest number of detected expressed proteins, 29 and 32, respectively. Frequently used abbreviations are presented in Abbreviations section, while the entire WCF protein profile for each strain is summated in Table S1. Lastly, a column graph summating the relative abundance values of acellular pertussis vaccine proteins (Figure 2) is presented.

### 3.2. Antibody Affinity Capture Identifies Known and Novel* Bp* Antigens

**Figure 3:**
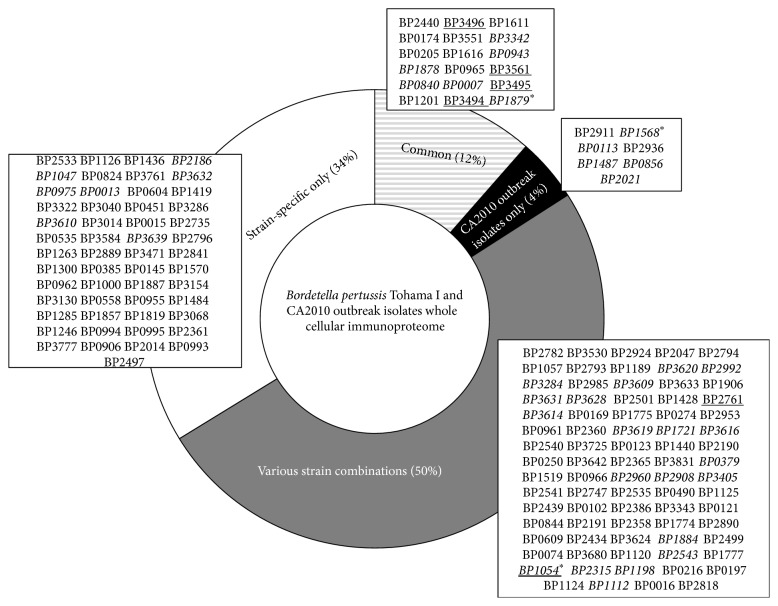
Schematic of* B. pertussis* Tohama I and CA2010 whole cellular immunoproteome identification distribution. Total number of putative and/or known immunoreactive proteins identified in the whole cellular fraction for each* Bp* strain or subsets (common—diagonal line; various: present in at least two strains—gray; only CA2010 isolates—black; specific strain—clear). Black asterisk represents acellular pertussis vaccine components: BP1879 (filamentous hemagglutinin), BP1568 (serotype fimbriae 3), and BP1054 (pertactin). Proteins previously showed immunoreactivity—italics (17-18); underlined (19).

After total WCF protein identification, each strain's immunoreactive potential was assessed by antibody affinity capture (IP) in parallel with nLC-ESI-MS/MS. A total of 157 proteins, known and putative* Bp* immunogenic proteins (PIPs), were identified with greater than 95% Scaffold (protein prophet) protein identification probability, of which 98% of this protein subset were commonly identified in all the strains WCF. Total identified antigens and PIPs captured (Figure 3; Table S2) from each individual* Bp* strain's WCF were primarily localized (Figure S2a) to the cytoplasm (55%), cytoplasmic membrane (8%), or periplasm (12%) according to PSORTb scores. Nearly 13% total combined proteins were localized to the outer membrane or were extracellular; two cellular localization sites generally associated with microbial pathogenesis and host antibody immune response. Additionally, the identified PIPs comprised traditional functions characteristic of antigens, such as adhesins and transporters (35–37), while key functions required for bacterial growth and survival including metabolism (24%), translation (25%) and transport (20%), are accounted for (Figure S2b). Of note, 47 of the 157 have been previously identified as PIPs, bacterial immunologs, or are known characterized* Bp* antigens including current acellular vaccine protein components FHA, Fim3, and Prn [17–20]. Eighteen, 7, 79, and 53 proteins were immunocaptured and categorized as commonly identified between the CA2010 and* Bp* T, CA2010 isolates only, various CA2010 stain combinations, or CA2010 strain specific, respectively.

## 4. Discussion

Several researchers have implemented genomic studies and analyzed allelic differences (especially in the genes encoding vaccine components) among* Bp* strain populations to support the hypothesis that circulating strains and the vaccine strain differ and that pathogen adaptation may contribute to pertussis resurgence. In 2013, King et al. [33] used genome-wide gene expression to profile Netherlands* Bp* isolates (*ptxP3* allele), revealing virulence factors, such as Ptx, had higher RNA transcript expression levels than the historic* ptxP1* strain. Such changes in circulating* Bp* isolates may lean towards enhanced* ptxP3* strain fitness and pertussis resurgence. In this study, we examined on a protein level CA* Bp* pertussis outbreak isolates as compared to the historic* Bp* T vaccine strain with an emphasis on acellular vaccine proteins (FHA, Ptx, Prn, and Fim 2/3).

### 4.1. Whole Cellular Proteome

Common and differential protein identification for these* Bp* isolates is determined by many factors including protein expression, MS/MS peptide detection, database searching, and any experimental variations. First, the 810 proteins commonly expressed between* Bp* T and the CA2010 comprise proteins, for instance, that are associated with energy production, metabolism, and translation machinery. Cell culture is a valuable experimental tool for* in vitro* cell growth but in principle is a mere surrogate for* in vivo* bacterial cell growth. Thus, virulent strains such as the CA2010 outbreak isolates may have differential growth and protein expression kinetics* in vitro* compared to the lab adapted strain* Bp* T. Absence of protein identification could simply indicate that the protein either was not synthesized, was lost in the initial protein extraction, or failed to be detected by MS/MS, due to amino acid composition resulting in a low ionization potential. Lastly, the CA2010 outbreak isolates have yet to be sequenced genetically; the database search is centered only on* Bp* T sequence information. Regardless of what determines protein identification, the protein profiles of these strains are insightful, especially for those proteins uniquely identified for the CA2010 isolates, as expression of these proteins may serve as indicators for virulence and immunogenicity.

### 4.2. Acellular Pertussis Vaccine* Bp* Proteins

Spectral counting (total number of tandem mass spectrum for an identified protein) has been used as a semiquantitative measure of relative abundance [31] and implemented in comparative proteomic studies (reviewed by Lundgren et al.) [32]. Since genetic variation among* Bp* virulent proteins has been observed in contemporary strains, a closer look at differential relative protein expression among the acellular vaccine proteins (represented by Scaffold normalized quantitative values) between* Bp* T and the CA2010 isolates is presented below. Importantly to make inferences regarding relative quantification, the strains examined in this study had comparable total protein quantities and were grown and harvested at similar optical densities.

FHA, a 368 kDa outer membrane protein plays a role in host cell binding and infection [34] is found in the* Bp* T WCF in greater relative abundance compared to the CA2010 isolates. Interestingly, the relative amount of FHA found in the culture supernatant (CS) is 3- to 6-fold higher in the CA2010 isolates compared to* Bp* T. FHA release into the extracellular milieu [35] is dependent on a maturated SphB1auto-transporter subtilisin-like protease [34, 36]. SpbH1 is also expressed in the WCF of each strain, with varying relative amounts, in which* Bp* 2 and* Bp* 5 have nearly a 1.5-fold increase compared to* Bp* T, suggesting lower levels of this protein in* Bp* T. This may explain the low FHA abundance in the* Bp* T CS.

Ptx, a five-subunit (S1 through S5) protein complex, was also identified in each WCF. The Ptx is a member of the A-B toxin family, where A consists of S1, a 33 kDa protein having ADP ribosylation activity, while B comprises S2 through S5 and is associated with host cellular adherence [34]. In the WCF, each Ptx subunit is found in varying amounts irrespective of the strain, except S4. Higher amounts of S4 were anticipated as this subunit is localized mainly to the cytoplasmic membrane, while the other subunits are primarily secreted. Additionally, S4 has been determined to be stoichiometrically present in the cell in a ratio twice as much as the other subunits [34] which was confirmed based on semiquantitative values. Furthermore in the CS protein fraction, S1 is present in much higher amounts (2.6- to 7.6-fold) in the CA2010 isolates compared to* Bp* T. S1 is a highly virulent protein subunit, as it has a major impact on host cell surface signaling with downstream intracellular events directly affecting immune cell response and host immune evasion [34]. Our relative quantitative Ptx S1 finding is partially in agreement with enzyme-linked immunosorbent assay studies which have shown that* ptxP3* alleles genetically identified in currently circulating* Bp* strains are associated with greater Ptx production [37]. One CA 2010 isolate carried the* ptxP1* allele and still presented a higher amount of Ptx S1 compared to* Bp* T. Importantly, Ptx is a major immunodominant protein in acellular vaccines. Higher level of Ptx S1 potentially being secreted by current* Bp* circulating isolates into the extracellular milieu presents an immunological challenge. Even though the acellular vaccine may have primed the host for heightened and proportionate response to S1, higher amounts of S1 in the cytosol may impede the innate immune stimuli resulting in suboptimal immunological priming. This has the propensity to lead to the production of low levels of S1 specific antibody resulting in residual-free biologically active S1 to continue the toxemic sequel.

**Figure 4:**
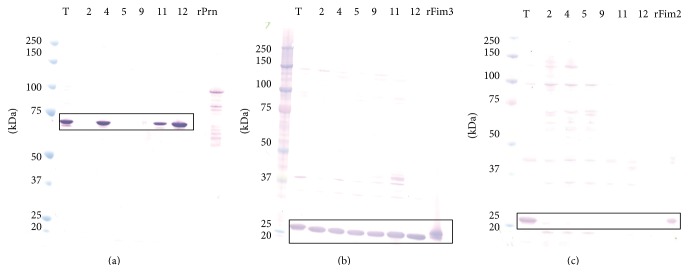
Western blot of* B. pertussis* isolates reveals deficiency in possible acellular pertussis vaccine protein components. (a) Prn, 69 kDa; (b) Fim3, 22.5 kDa; and (c) Fim2, 22 kDa. T—*Bp* Tohama I;* Bp* CA2010 isolates: 2, 4, 5, 9, 11, and 12. Recombinant (r) proteins were used as western blot controls, and all blue or dual color protein molecular weight markers for size standards. The black box highlights the protein of interest.

Prn, a 69 kDa autotransporter which adheres to host cells [34, 38], was differentially expressed among the CA2010 isolates while failing MS/MS detection in* Bp* 9. Semiquantitative values revealed very low relative levels of Prn for* Bp* 2 and* Bp* 5 compared to* Bp* T. Western blot analysis further revealed and confirmed pertactin deficiency in* Bp* 2,* Bp* 5, and* Bp* 9 (Figure 4(a)). In general, Prn-negative findings are not improbable, as, recently, several* Bp* strains isolated from children in Pennsylvania (US) were sequenced and revealed that either an insertion sequence disrupted the* prn* protein coding region or there was an amino acid substitution that resulted in a truncated Prn protein [39]. Moreover, Pawloski et al. [16] described a genetic characterization of a historic collection of* Bp* isolated in the US revealing* prn*-deficiency, with high prevalence between the years 2010 to 2012. As a component of acellular vaccines, upon administration, human immune responses are primed to recognize Prn, so* Bp* isolates failing to express a native full-length protein could have immunological implications such as modified epitope recognition, reduced antibody neutralization, or inefficient phagocytosis of* Bp* by host immune cells [34, 39].

Serotypes Fim 2 and Fim 3, cell surface-exposed and extracellular proteins, likely involved in the attachment of* Bp* to host cells [34], play a very important first step for pertussis infection. Also, the proteins are components of some acellular vaccines. Fim 3 is commonly identified in the WCF and CS of all of the CA2010 isolates as well as* Bp* T. Notably, the relative amount of Fim3 in the* Bp *T WCF was at least 10-fold lower compared to the CA2010 isolates, giving a possible reason why there are very low detectable levels in the* Bp* T CS. Immunoreactive banding patterns associated with Fim3 was evident in all 7 strains (Figure 4(b)). Moreover, Fim 2 was detected in the WCF of only* Bp* T,* Bp* 2,* Bp* 4, and* Bp* 12. There was about 66-, 27-, and 66-fold reduction of Fim 2 concentration in* Bp* 2,* Bp* 4, and* Bp* 12, respectively, compared to* Bp* T WCF. Conversely in the CS, Fim 2 was identified only in* Bp* T and* Bp* 2. Even more, western blot analysis revealed markedly reduced immunodetection of Fim2 among the Fim2 positive CA2010 isolates, while highly immunoreactive in* Bp* T (Figure 4(c)). Hypothetically, subtle changes in the amino acid sequence of the chaperone and usher proteins required for fimbrium biogenesis (FimB, FimC, and FimD) [34] may have possibly led to diminished functional capacities to properly sort Fim2 and Fim3 to the outer-membrane and/or release in the extracellular environment. Or, with respect to Fim2, the isolates may have simply evolved and no longer possess the fim2 locus or there are insertions and/or deletions in the gene in which the protein is no longer expressed or is truncated yielding structural and functional abnormalities. In both instances, a more thorough examination of these proteins would need to be performed at the genetic level.

The aforementioned acellular vaccine protein components and effector proteins (i.e., SpbH1, FimB, FimC, and FimD) identified in each strain are all a reflection of protein expression. The expressions of most* Bp* virulent proteins are under the regulation of a two-component regulatory system, BvgS and BvgA, occurring mainly in a high BvgA^+^ mode of expression [34]. In response to environmental signals, like temperature,* Bp* is able to modulate the expression of these proteins for necessity, survival, and further pathogenesis to host cells* in vivo*.* In vitro* bacterial cell culture is informative but is limited as bacterial growth (35°C) and subsequent protein expression merely mimics* in vivo* bacterial-host cell processes. Growing the CA2010 isolates in the presence of human tracheal epithelial cells, for instance, eliciting a true cellular induction, followed by bacterial protein extraction and nLC-ESI-MS/MS protein identification, would provide a greater biological understanding. Also, infecting mice with the CA2010 isolates, monitoring the* Bp* colonization, and infection phases over time using bioluminescence approaches, followed by relevant host tissue removal,* Bp* protein extraction and proteomic examination may provide an* in vivo* perspective. Importantly, the relative amounts of these virulence factors present in the WCF, CS, or tissue culture system could be measured in more absolute terms using quantification methods such as isotope-dilution mass spectrometry [40].

### 4.3. Immunoproteome

The identification and relative expression of virulence proteins associated with pertussis outbreak isolates are important as they could provide insight on how these recent circulating isolates mechanistically cause disease. Equally important are the proteins that invoke an immune response, as these proteins, once validated, could potentially serve as candidates for improved pertussis vaccines. Strain specific antiserum against each clinical CA2010 isolate was not available, so a rabbit polyclonal serum generated against intact whole cell* Bp* T served as a surrogate to assess each CA2010 isolates immune potential. Antibody affinity pull-down studies revealed the identification of a collective total 157 known and putative antigens captured from the WCF of each CA2010 isolate.

Despite the fact that nearly 98% of the antigens/PIPs identified in the IP were commonly found in each WCF, only about 10% of the immune proteins detected are common in all of the strains, suggesting extensive antigenic diversity even among strains drawn from the same epidemic. Patient demographics, vaccine, and immune status may have been major contributing factors for this antigenic variation. The scantiness of immune proteins in these strains may also be due to the limited antigen capture efficacy in the immunoproteome analysis. The immunoproteome analysis is primarily driven by the antibody diversity in the* Bp* T capture polyclonal antiserum. The* Bp* T, a lab adapted strain, has diminished immunogenicity possibly due to the lack of selection pressure and continued* in vitro* passage. Even though this strain is grown and harvested in standardized culture conditions similar to clinical strains used in this study, this diminished immunogenicity in* Bp* T may result in variable antigen expression* in vivo*. This variable immunogenicity yields to limited antibody repertoire in the polyclonal antiserum. This is further compounded by the possibility of variable levels of expression of these antigens* in vivo* leading to fluctuation in the quantity and quality of antibodies in the rabbit serum. These limitations in the antibody repertoire in polyclonal antiserum are likely to have a direct impact on the number of proteins captured in each WCF. While limited antibody repertoire narrows the immunoproteome detection spectrum in WCF, difference in specific antibody concentration impacts its detection sensitivity due to changes in the propensities of stable antigen-antibody complex. For instance, antigens/PIPs exclusive to the CA2010 (i.e., Fim3) have been shown to be antigenic, yet there appeared to be no antigen-antibody interaction in* Bp* T even though homologous* Bp* T rabbit antiserum was used as “bait” in the capture. Moreover, since the WCF used in the capture are in essence a soluble protein lysate, some of these proteins may have lost their native conformational state, in which the epitopes ultimately may not be recognized by antibodies in the pool. In addition to the aforesaid immunological reasoning, poor ionization and/or peptide masking due to other abundant proteins in the sample mixture may have impacted the MS detection sensitivity leading to lowered immunoproteome output.

It would be surprising and rather difficult to explain if the CA2010 strains WCF exhibited homologous immunoproteome profiles with* Bp* T polyclonal serum. As anticipated, these isolate's PIPs are highly heterogenic especially in their reactivity to* Bp* T serum. This observation in itself clearly indicates changes in the* Bp* virulence and antigenic profile over time and the need for similar periodical immunoproteome analysis using serum generated with the vaccine strain.

## 5. Conclusion

With increased pertussis cases in recent years, it is imperative to have an understanding of both the genetic and the proteomic profiles of these circulating* Bp* isolates. Acellular vaccine-induced immunity in the present time is generated in response to proteins purified from a* Bp* strain that was isolated more than 50 years ago. As suggested in this study, newer* Bp* isolates in circulation have variable expression kinetics of vaccine components (i.e., Prn and Ptx S1) compared to the historic* Bp* T. The very fact that the clinical isolates have very limited antigenic homogeneity with the* Bp* T explains the biological basis for poor immune protection with acellular vaccines. These shifts will likely have ramifications on how a primed human immune system protects against* Bp* colonization and infection. To be more representative of strains that exist in the environment today, and notwithstanding manufacturing constraints, current acellular vaccines could be supplemented with proteins displaying high or higher antigenicity or even more to be proactive consider the design of a novel more protective efficacious vaccine. Antibody affinity capture technologies implemented here identified putative antigens from* Bp* clinical isolates, CA2010. Strain specific human serum from active infection or a pooled convalescent human serum would provide a greater immune appreciation and should be considered for future proteomic* Bp* outbreak strain assessments. Importantly, more* Bp* strains from this 2010 CA outbreak as well as other pertussis US outbreaks/epidemics [41] will need to be examined at the genotype and proteome level to draw any significant inferences regarding possible increased virulence protein expression among the circulating isolates and any correlation with strain fitness, pertussis resurgence in the US, and impact on vaccine-induced immunity. Animal studies, such as the baboon model [42], examining proteomic changes related to* Bp* colonization, transmission, pathogenicity, and immune response using recent* Bp* isolates plus/minus acellular pertussis vaccination may be considered as a long-term future endeavor.

This proteomic study has laid the foundation for the use of mass spectrometric methods that rapidly detect the presence of expressed virulence proteins, such as FHA or Ptx, in various subcellular matrices. Collectively these methods could be used as supplementary tools with current and traditional pertussis diagnostic tools, aiding in rapid* Bp* strain differentiation, diagnosis, treatment, and prevention of pertussis.

## Supplementary Material

Table S1 represents a list of the total whole cellular proteome (acid-soluble protein profile) identified among Bordetella pertussis (Bp) Tohama I (T) and the Bp CA2010 outbreak isolates (Bp 2, 4, 5, 9, 11 and 12). Proteins were identified using nano-liquid chromatography-tandem mass spectrometry (nLC-MSMS), database searching and validated using Scaffold (Proteome Software). Information such as Gi accession number, molecular weight, subcellular localization, gene function, gene name, amino acid coverage and number of unique peptides are provided in the table. Table S2 represents a list of the total immunoproteome (a known and putative antigenic protein profile) identified among Bp T and the Bp CA2010 outbreak isolates (Bp 2, 4, 5, 9, 11 and 12). The proteins were identified using antibody affinity capture technologies in parallel with nLC-MSMS, database searching and Scaffold validation. As in the case for table S1, relevant information pertaining to the protein is provided. Figure S1 is a schematic summation of subcellular localization (a) and gene ontology (b) associated with the proteins identified in the whole cellular proteome. Figure S2 is a schematic summation of subcellular localization (a) and gene ontology (b) associated with the proteins identified in the immunoproteome. Lastly, a detailed legend including abbreviations and references cited are provided for each supplementary table or figure.

## Figures and Tables

**Table 1:** Whole cellular fraction protein profiles of *B. pertussis* California 2010 outbreak isolates identified by nLC-MS/MS.

	T	2	4	5	9	11	12
Common	814	814	814	814	814	814	814
Various	274	293	259	208	208	208	210
CA2010 only	0	27	27	27	27	27	27
Strain specific	29	32	6	7	4	10	10
Total	**1117**	**1166**	**1106**	**1056**	**1053**	**1059**	**1061**

A numerical summary of the total number of proteins identified in the whole cellular fraction for each *Bp* strain or subsets (common, various: present in at least two strains, CA2010 isolates only, or identified in a specific strain). Protein identification is based on greater than 95% Scaffold protein identification probability. *Bp* Tohama I - T; *Bp* CA2010 isolates: 2, 4, 5, 9, 11, and 12.

## Abbreviations

*Bp*: * Bordetella pertussis*
T: Tohama I
CA2010: California 2010 outbreak isolates
nLC: Nanoflow liquid chromatography
ESI: Electrospray ionization
MS/MS: Tandem mass spectrometry
WCF: Whole cellular fraction (soluble protein extracts)
CS: Culture supernatant
PIPs: Putative immunogenic proteins
Ptx S1 to S5: Pertussis toxin subunits 1 through 5
FHA: Filamentous hemagglutinin
Fim2: Serotype fimbriae 2
Fim3: Serotype fimbriae 3
Prn: Pertactin
BvgA: Virulence regulatory expression system.
